# Supplementary figures and images for: Tree and shrub richness modifies subtropical tree productivity by regulating the diversity and community composition of soil bacteria and archaea
Source: Microbiome. 2023 Nov 23;11:261. doi: 10.1186/s40168-023-01676-x (PMC10666335; doi:10.1186/s40168-023-01676-x)

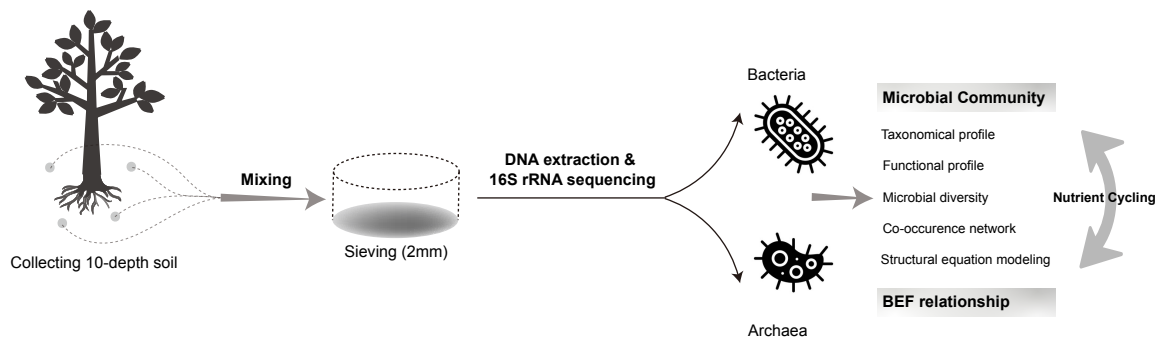

Supplement: Supplementary file 2 — Additional file 1: Figure S1. Flow chart of sampling, DNA extraction, microbial sequencing, and the detailed information of dissecting microbial community driven BEF relationships in a subtropical forest. [file 40168_2023_1676_MOESM1_ESM.pdf]

**a**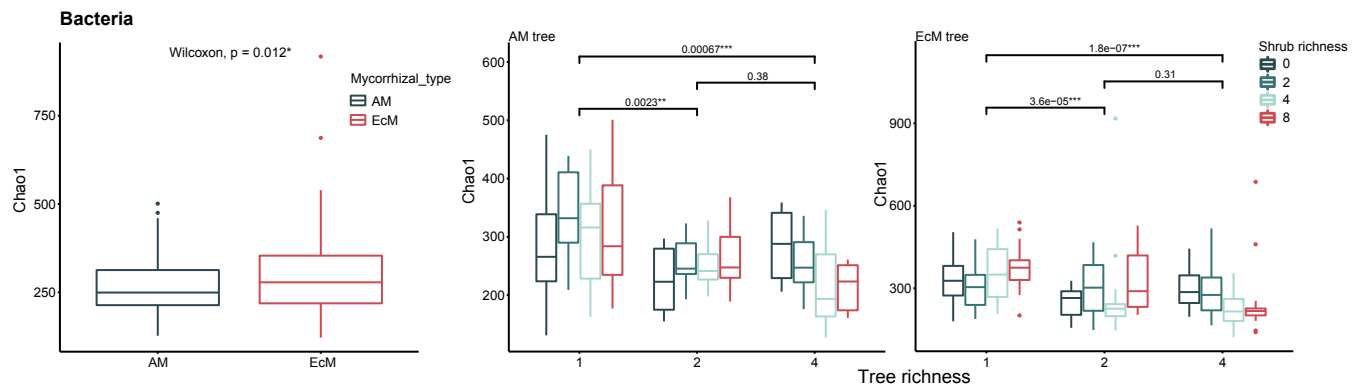**b**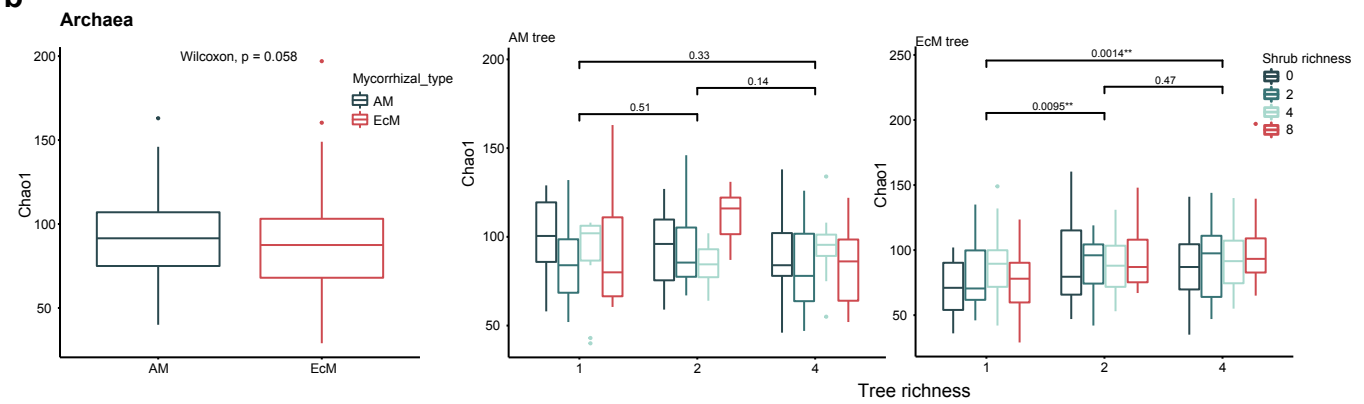

Supplement: Supplementary file 4 — Additional file 3: Figure S3. Soil bacterial and archaeal α-diversity under three tree species richness levels (1, 2, and 4) and four shrub species richness (0, 2, 4, and 8), respectively for both ectomycorrhizal fungi-colonized trees (EcM) and arbuscular fungi-colonized trees (AM). [file 40168_2023_1676_MOESM3_ESM.pdf]

**a**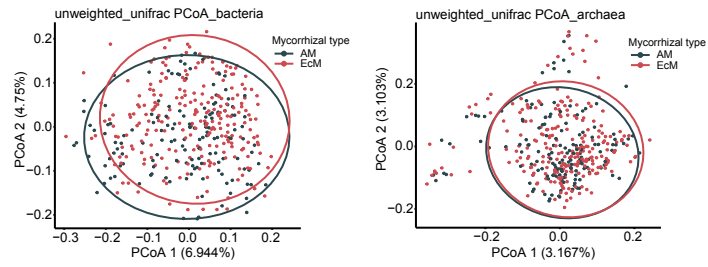**b**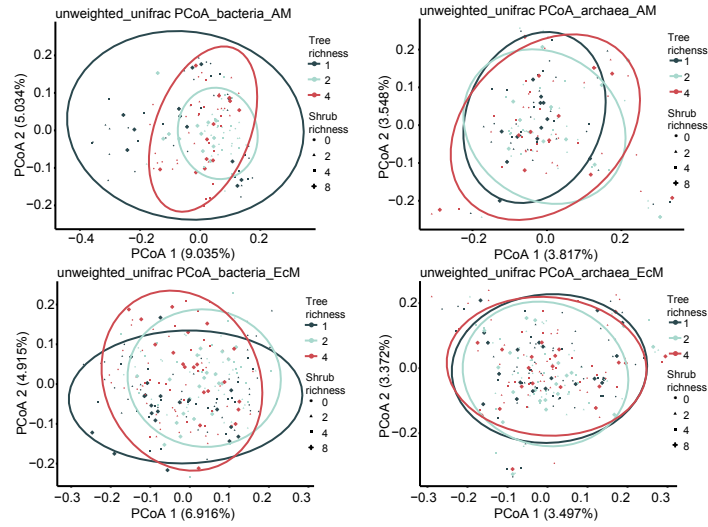

Supplement: Supplementary file 5 — Additional file 4: Figure S4. Principal coordinates analysis (PCoA) with unweighted unifrac distances matrices to visualize the bacterial and archaeal community composition for ectomycorrhizal fungi-colonized trees (EcM) and arbuscular fungi-colonized trees (AM). a. the effect of mycorrhizal types on community compositions of bacteria and archaea. b. the combined effects of tree species richness and shrub species richness on community compositions of bacteria and archaea, respectively for ectomycorrhizal fungi-colonized trees (EcM) and arbuscular fungi-colonized trees (AM). [file 40168_2023_1676_MOESM4_ESM.pdf]

**a**

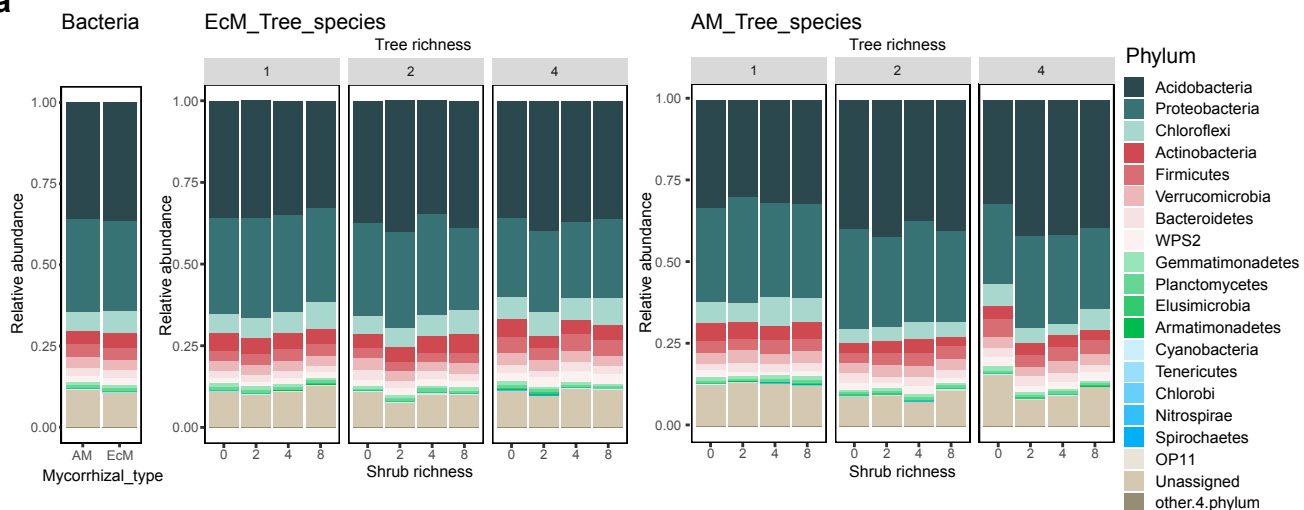

**b**

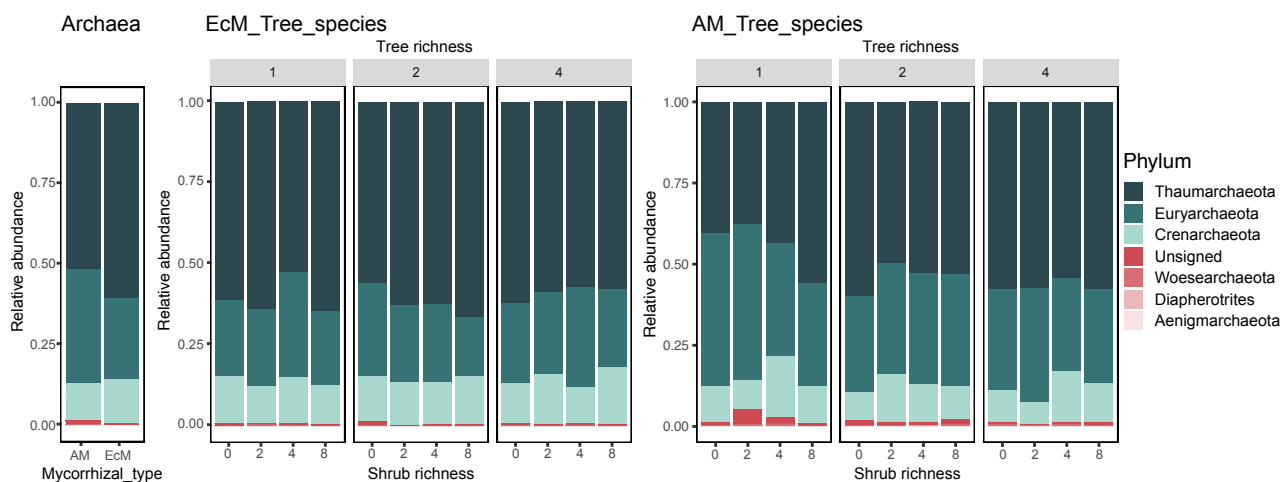

Supplement: Supplementary file 6 — Additional file 5: Figure S5. Taxonomic classifications of soil bacterial and archaeal community for ectomycorrhizal fungi-colonized trees (EcM) and arbuscular fungi-colonized trees (AM). [file 40168_2023_1676_MOESM5_ESM.pdf]

## Bacteria

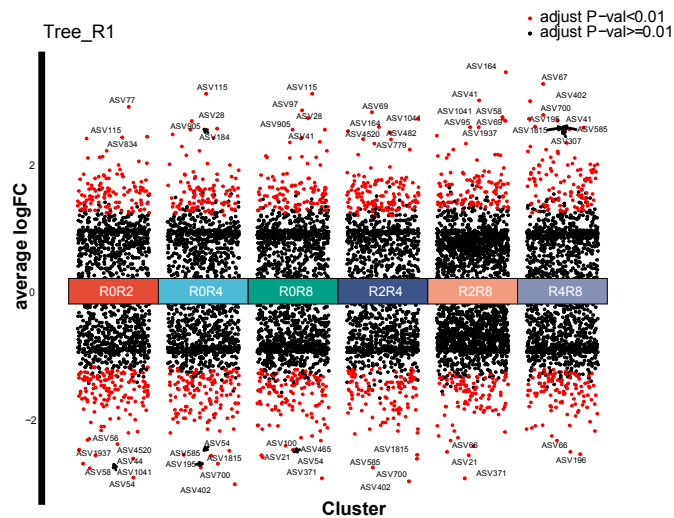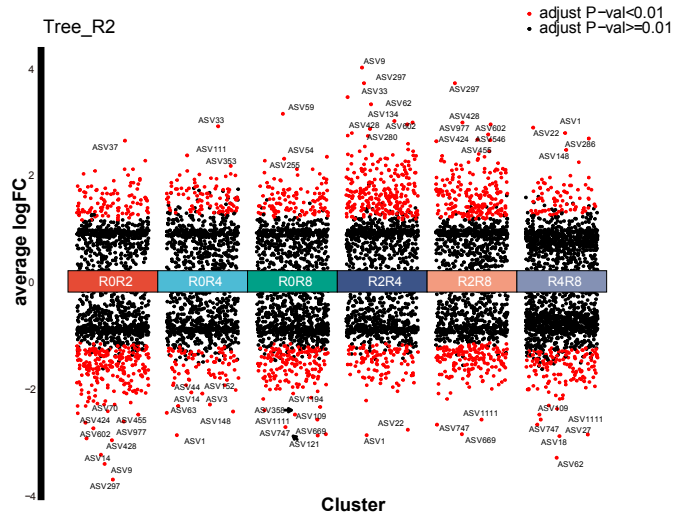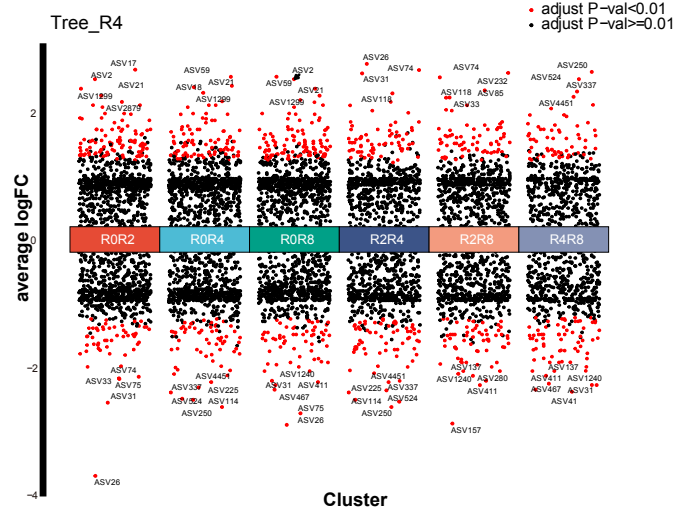

## Archaea

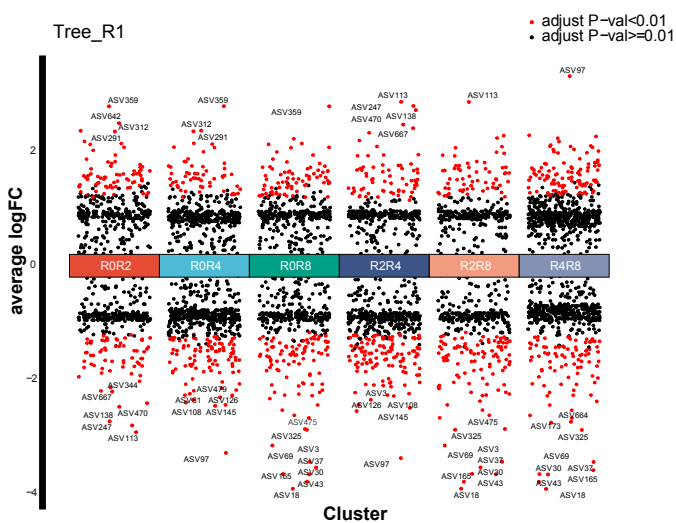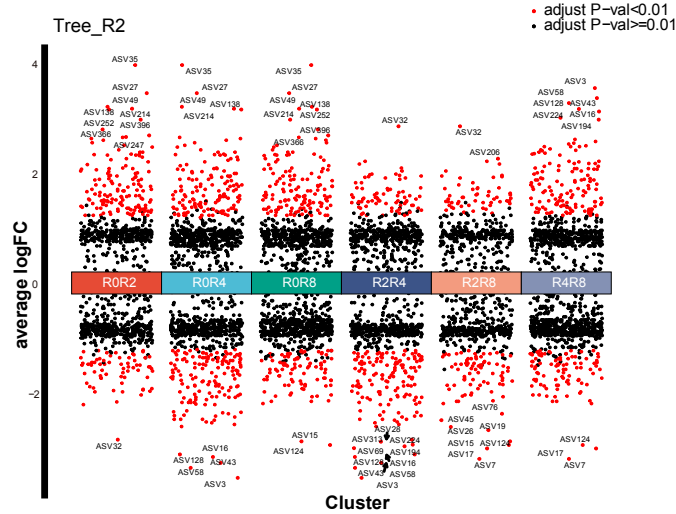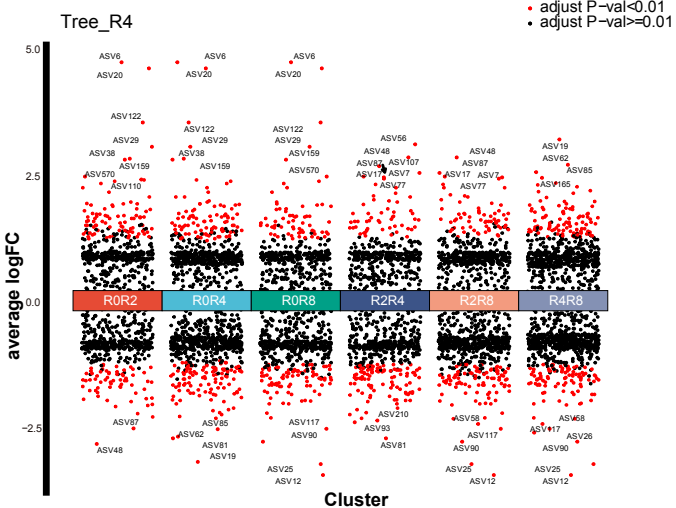

Supplement: Supplementary file 7 — Additional file 6: Figure S6. Volcano plots showing up- and down-regulated ASVs in six comparisons between shrub richness levels at 0, 2, 4, 8 (R0R2, R0R4, R0R8, R2R4, R2R8, R4R8) under three tree species richness levels, respectively for bacteria and archaea. An adjusted p value < 0.01 is indicated in red, while an adjusted p value < 0.01 is indicated in black. The top ten ASVs with the most significant differences in abundance were indicated by their ID numbers and the numbers of ASVs with significantly differences in abundance for the three comparisons are indicated in bracket. [file 40168_2023_1676_MOESM6_ESM.pdf]

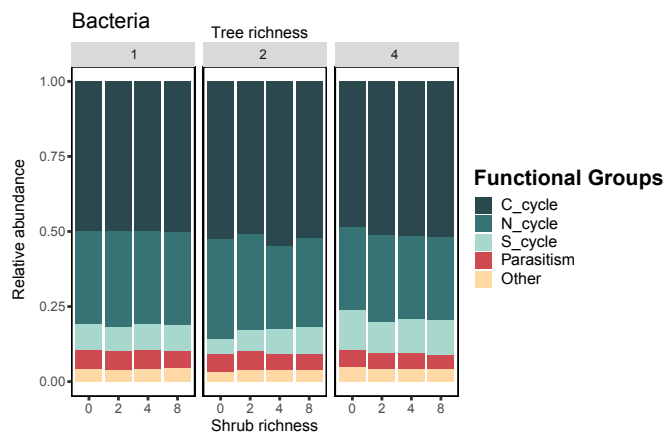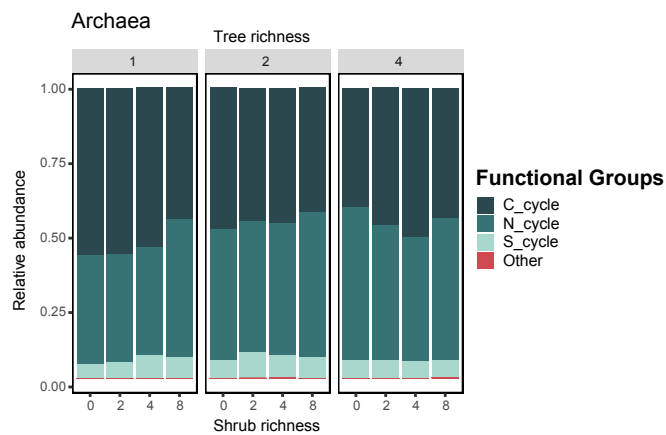

Supplement: Supplementary file 8 — Additional file 7: Figure S7. Functional assignments with relative abundance of each functional groups in bacterial and archaeal community, including carbon cycling (C_cycle), nitrogen cycling (N_cycle), sulfur cycling (S_cycle), parasitism and others under the combined effects of tree species richness and shrub species richness. [file 40168_2023_1676_MOESM7_ESM.pdf]

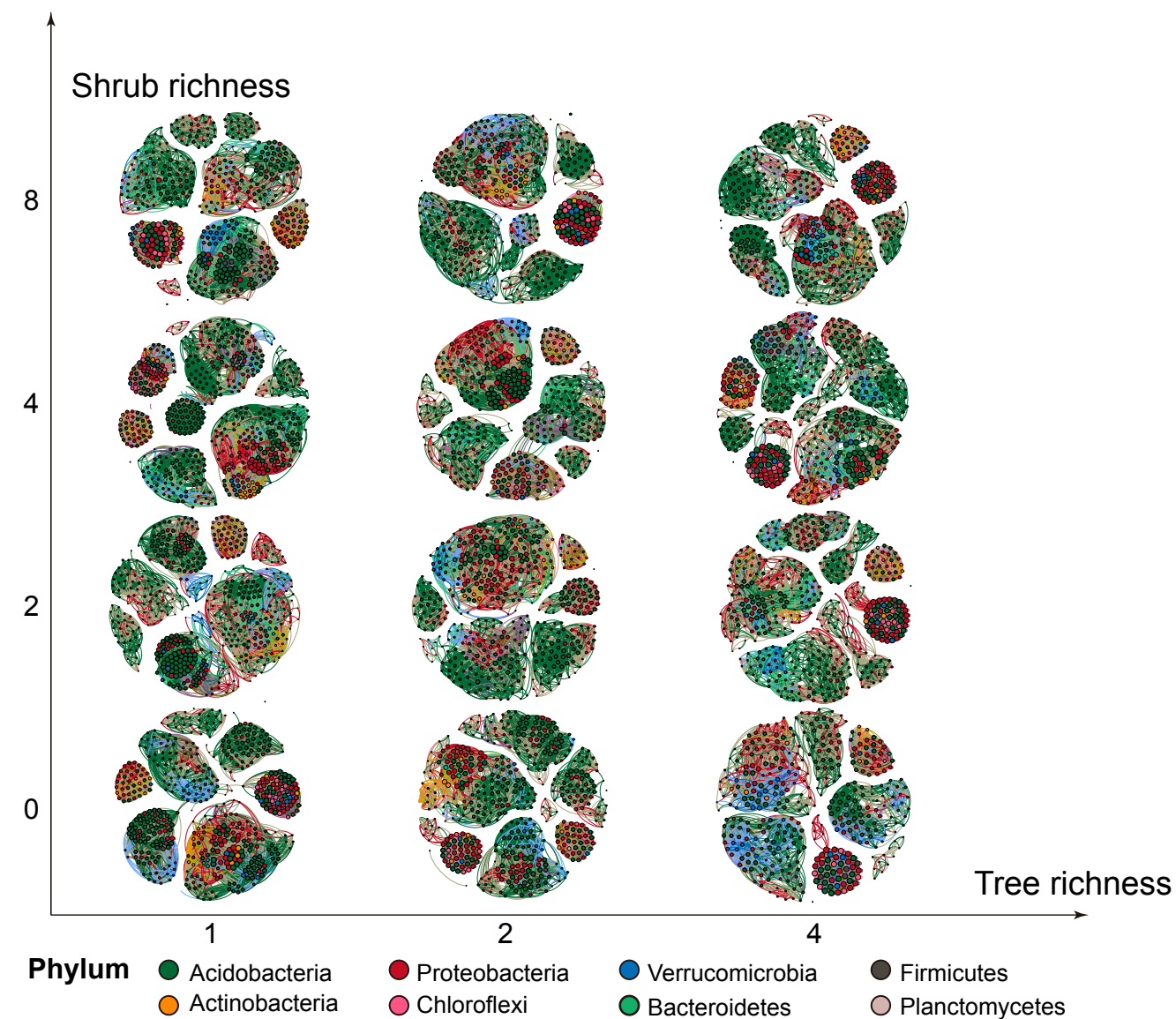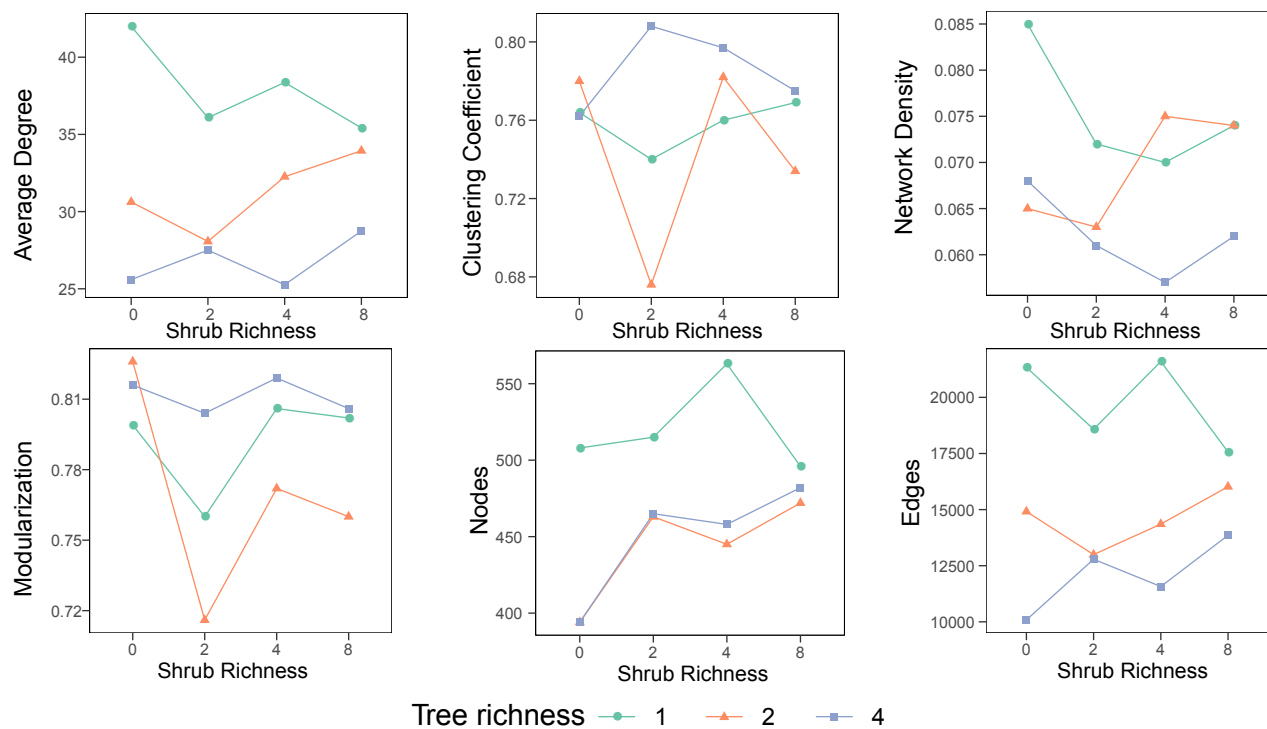

Supplement: Supplementary file 9 — Additional file 8: Figure S8. The co-occurrence networks of bacterial communities in three tree species richness levels (1, 2, and 4) coupled with four shrub species richness levels (0, 2, 4, and 8). The nodes in the networks are colored referred to the taxonomic assignments at phylum level and the size of each node is proportional to the relative abundance. [file 40168_2023_1676_MOESM8_ESM.pdf]

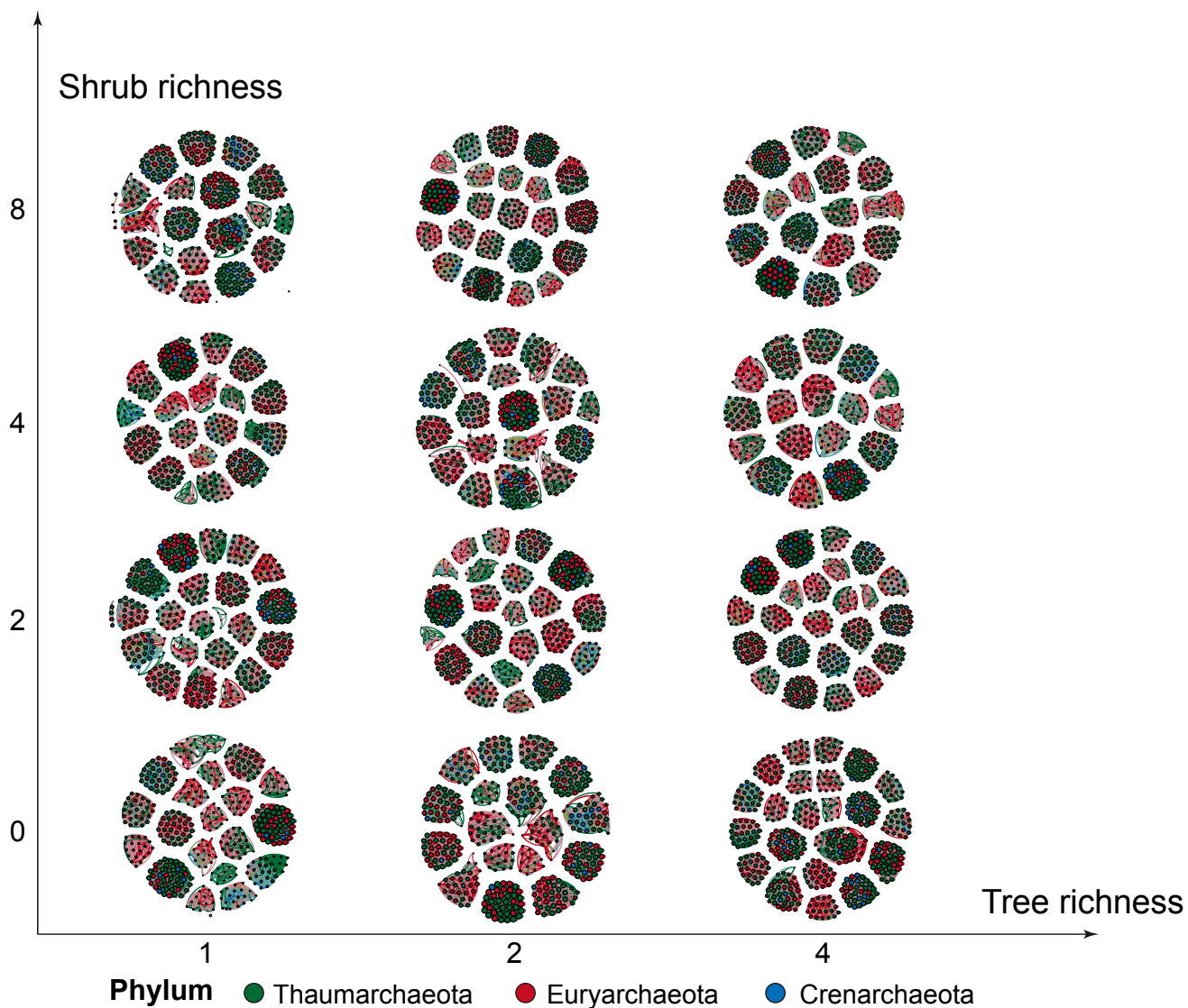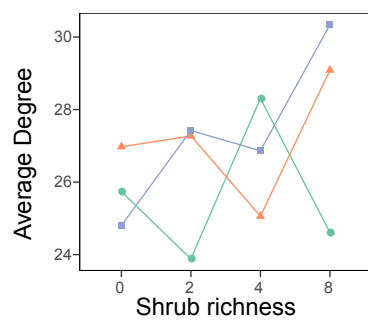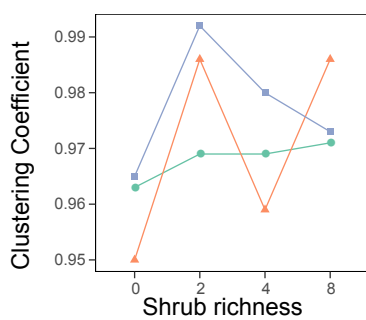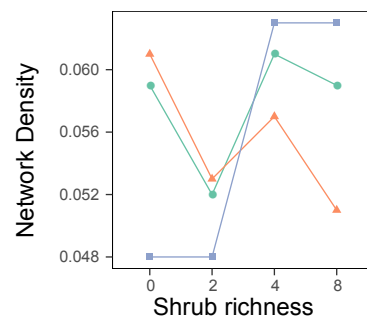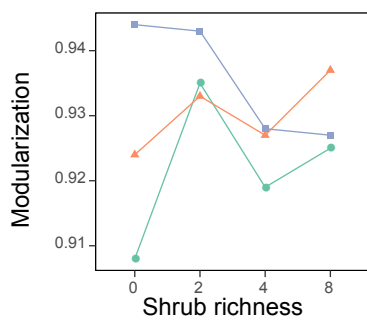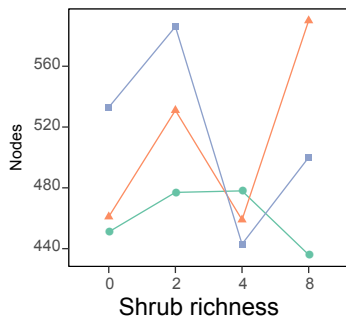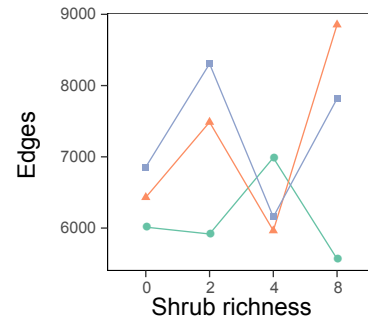

Tree richness — R1 — R2 — R4

Supplement: Supplementary file 10 — Additional file 9: Figure S9. The co-occurrence networks of archaeal communities in three tree richness levels (1, 2, and 4) coupled with four shrub richness levels (0, 2, 4, and 8). The nodes in the networks are colored by the taxonomic assignments at phylum level and the size of each node is proportional to the relative abundance. [file 40168_2023_1676_MOESM9_ESM.pdf]

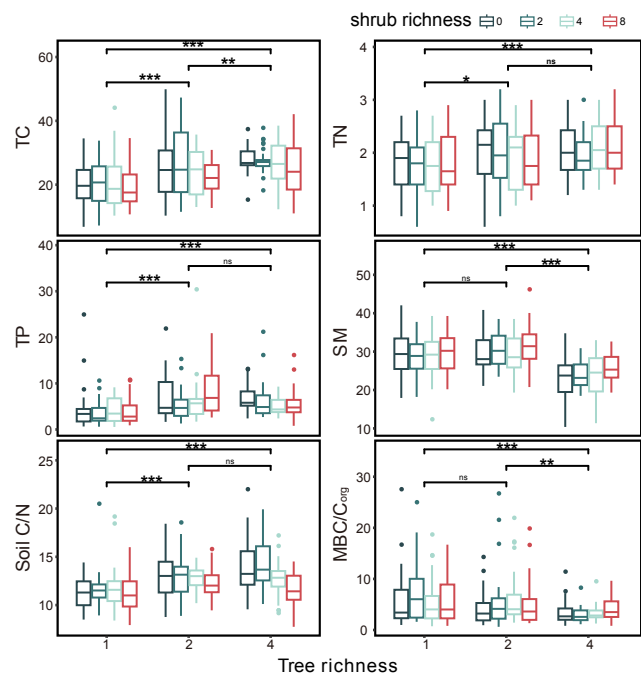

Supplement: Supplementary file 11 — Additional file 10: Figure S10. Direct effects of aboveground plant species richness on environmental factors. [file 40168_2023_1676_MOESM10_ESM.pdf]
