# Supplementary material for: Tree and shrub richness modifies subtropical tree productivity by regulating the diversity and community composition of soil bacteria and archaea
Source: Microbiome. 2023 Nov 23;11:261. doi: 10.1186/s40168-023-01676-x (PMC10666335; doi:10.1186/s40168-023-01676-x)
Supplement: Supplementary file 3 — Additional file 2: Figure S2. A priori structural equation modeling (SEM) hypothesized causal pathways of how tree/shrub species richness and soil properties may influence stand-level tree productivity through modifying the bacterial and archaeal communities and microbial biomass carbon or nitrogen content. [file 40168_2023_1676_MOESM2_ESM.pdf]

Conceptual model

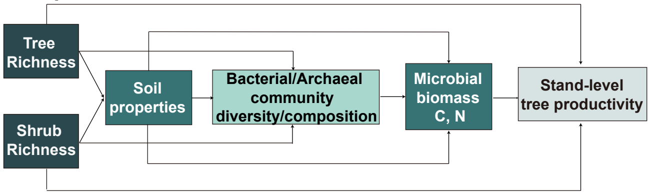

Hypothesis model 1

Bacteria

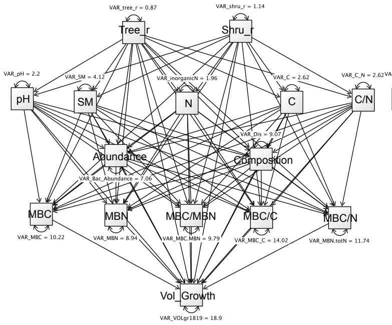

RMSEA = 0.025; CFI = 0.998; SRMR = 0.046

Archaea

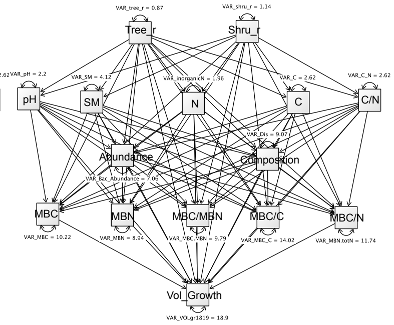

RMSEA = 0.061; CFI = 0.99; SRMR = 0.059

Hypothesis model 2

Bacteria

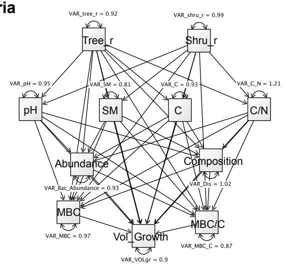

RMSEA = 0; CFI = 1.005; SRMR = 0.019

Archaea

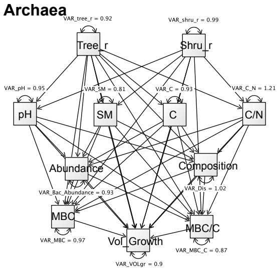

RMSEA = 0.306; CFI = 0.765; SRMR = 0.11

Hypothesis model 3

Bacteria

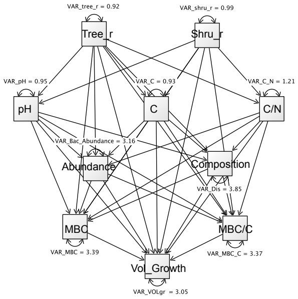

RMSEA = 0.332; CFI = 0.843; SRMR = 0.028

Archaea

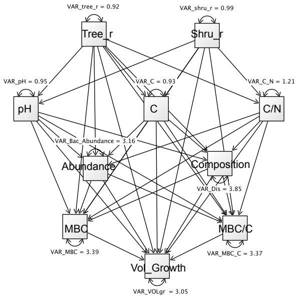

RMSEA = 0.382; CFI = 0.687; SRMR = 0.154

Hypothesis model 4

Bacteria

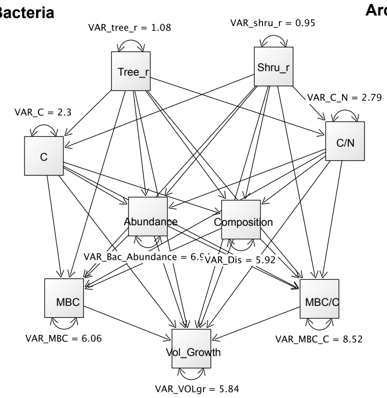

RMSEA = 0; CFI = 1.001; SRMR = 0.036

Archaea

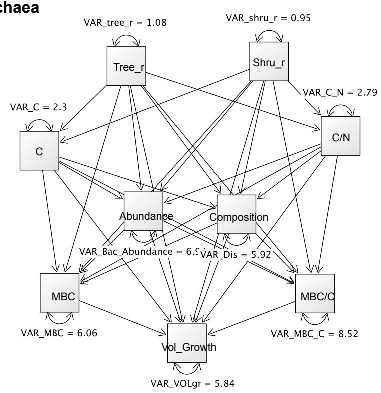

RMSEA = 0.481; CFI = 583; SRMR = 0.233
